# Social, economic, and health impact of the respiratory syncytial virus: a systematic search

**DOI:** 10.1186/s12879-014-0544-x

**Published:** 2014-10-30

**Authors:** Javier Díez-Domingo, Eduardo G Pérez-Yarza, José A Melero, Manuel Sánchez-Luna, María Dolores Aguilar, Antonio Javier Blasco, Noelia Alfaro, Pablo Lázaro

**Affiliations:** Centre of Public Health Research of Valencia-FISABIO, Valencia, Spain; Division of Pediatric Respiratory Medicine, Hospital Universitario Donostia-Instituto Biodonostia, San Sebastián, Spain; Biomedical Research Centre Network for Respiratory Diseases (CIBERES), San Sebastián, Spain; Department of Pediatrics, University of the Basque Country (UPV/EHU), San Sebastián, Spain; National Center of Microbiology and CIBER of Respiratory Diseases, Instituto de Salud Carlos III, Madrid, Spain; Neonatology Division, Hospital General Universitario Gregorio Marañón, Madrid, Spain; Advanced Techniques in Health Services Research, Madrid, Spain

**Keywords:** Respiratory syncytial virus, Social impact, Health impact

## Abstract

**Background:**

Bronchiolitis caused by the respiratory syncytial virus (RSV) and its related complications are common in infants born prematurely, with severe congenital heart disease, or bronchopulmonary dysplasia, as well as in immunosuppressed infants. There is a rich literature on the different aspects of RSV infection with a focus, for the most part, on specific risk populations. However, there is a need for a systematic global analysis of the impact of RSV infection in terms of use of resources and health impact on both children and adults. With this aim, we performed a systematic search of scientific evidence on the social, economic, and health impact of RSV infection.

**Methods:**

A systematic search of the following databases was performed: MEDLINE, EMBASE, Spanish Medical Index, MEDES-MEDicina in Spanish, Cochrane Plus Library, and Google without time limits. We selected 421 abstracts based on the 6,598 articles identified. From these abstracts, 4 RSV experts selected the most relevant articles. They selected 65 articles. After reading the full articles, 23 of their references were also selected. Finally, one more article found through a literature information alert system was included.

**Results:**

The information collected was summarized and organized into the following topics: 1. Impact on health (infections and respiratory complications, mid- to long-term lung function decline, recurrent wheezing, asthma, other complications such as otitis and rhino-conjunctivitis, and mortality; 2. Impact on resources (visits to primary care and specialists offices, emergency room visits, hospital admissions, ICU admissions, diagnostic tests, and treatments); 3. Impact on costs (direct and indirect costs); 4. Impact on quality of life; and 5. Strategies to reduce the impact (interventions on social and hygienic factors and prophylactic treatments).

**Conclusions:**

We concluded that 1. The health impact of RSV infection is relevant and goes beyond the acute episode phase; 2. The health impact of RSV infection on children is much better documented than the impact on adults; 3. Further research is needed on mid- and long-term impact of RSV infection on the adult population, especially those at high-risk; 4. There is a need for interventions aimed at reducing the impact of RSV infection by targeting health education, information, and prophylaxis in high-risk populations.

**Electronic supplementary material:**

The online version of this article (doi:10.1186/s12879-014-0544-x) contains supplementary material, which is available to authorized users.

## Background

The respiratory syncytial virus (RSV) causes acute bronchiolitis (AB) and upper and lower respiratory tract infections. The risk of severe AB, and related complications, caused by RSV, is well-known among preterm infants, those with severe congenital heart disease (CHD), bronchopulmonary dysplasia (BPD), malformations, neuromuscular diseases and immunological disorders. AB is the infection of the lower respiratory tract most commonly found among newborns [1],[2]. The annual incidence of AB in newborns is 10% [3] with an admission rate between 2 and 5%, which has seen a substantial increase in recent years [3],[4]. AB risk is increasing in many clinical situations, especially in the presence of immunosuppression. For instance, among children, those with Down syndrome [5], cystic fibrosis [6], biliary atresia [7], or transplant patients [8] are at higher risk for complications or severe forms of the disease than the general population. Further, among transplant patients, the risk of organ rejection is greater among children with AB than those without the condition [8]. However, despite the increased risk of these pathologies, we are not aware of a systematic study capturing the extent and relevance of their potential impact in terms of health as well as social and healthcare resources required.

In adults, although RSV infection was identified with pneumonia in the 1960s, we only started to comprehend its importance on adult respiratory infections two decades ago [9]-[12]. In fact, some studies suggest that the clinical impact of RSV in some adult risk populations may be similar to that of the seasonal influenza virus [13],[14]. These risk populations include cardiorespiratory disease patients, frail elderly, and severely immunosuppressed patients [10],[11],[15],[16]. Unlike pediatric literature, there is an important gap in knowledge about the epidemiology of RSV infection in adult literature, [17] though the case may be that available data on RSV infection impact are scattered among publications in different fields.

Thus, we designed the current study with the main objective of determining the social, economic, and health impact of RSV infection on children and adults based on the best available scientific evidence.

## Methods

The study was designed to start with a systematic search of scientific literature performed by an archivist specialized in medical research. Then, an expert in critical reading carried out a first screening of the citations identified based on titles and abstracts. Selected abstracts were then examined by 4 experts (two public health specialists, one neonatologist, and one pediatric pulmonologist) who decided on the articles most relevant to the study aims.

### Systematic search

The systematic search of literature examining the RSV social, economic, and health impact was performed during the month of September, 2012 within specific parameters regarding publication year, language (English or Spanish), field of study, population of interest, and type of publication.

The following engines were searched for the periods specified: MEDLINE from 1950 on, EMBASE from 1974 on, Spanish Medical Index (IME for its acronym in Spanish) from 1970 to 2006, MEDES-MEDicina in Spanish from 2001 on, and the latest available issue of the Cochrane Plus Library (Number 1, 2012). To avoid missing any articles published in Spanish journals not indexed in IME or MEDES, we performed a search using Google search engine and we reviewed the first 100 results returned. Terms for these database searches included keywords closely matching the relevant medical field headings: *respiratory syncytial virus*, and *respiratory syncytial pneumovirus*.

We designed this non-specific but highly sensitive search to avoid missing any relevant information. The delimitation of the search was performed *a posteriori* by selecting titles and abstracts that included information on: a) incidence; b) impact on health (respiratory infections, asthma, mortality, otitis media, apnea, neurological involvement, cardiac involvement, and chronic obstructive pulmonary disease (COPD); c) impact on healthcare resources (hospital admissions, intensive care unit (ICU) admissions, diagnostic tests, and treatments; d) impact on direct and indirect costs; e) and strategies to reduce the impact, such as hygienic measures and prophylactic treatments.

### Title selection

The searches identified 10,146 references of which 3,548 were duplicate titles, resulting in 6,598 references. Articles were first filtered by discarding those with no information on RSV's impact. This selection reduced the number of references of interest to 876. Based on a reading of these 876 abstracts, 421 were selected.

### Article selection

**Figure 1:**
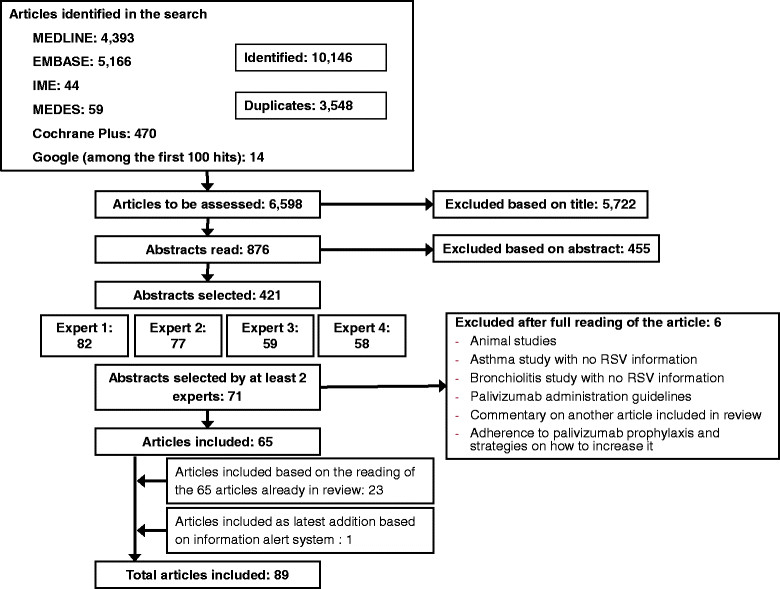
**Flow chart of study selection process.** RSV: respiratory syncytial virus. IME: Spanish Medical Index (for its acronym in Spanish).

The 421 abstract selected were sent to 4 experts with experience in clinical practice, research, and epidemiology relative to RSV. They were asked to select articles using as inclusion criteria the social, economic, and RSV health impact relevance. The four experts selected 82, 77, 59, and 58 articles, respectively. From these only those 71 articles selected by at least 2 experts were included. Next, based on the reading of the full articles, 6 were excluded for lack of relevance to the aim of the study. In addition to the 65 articles [18]-[82], 23 more citations found in the reference lists of these 65 articles were included for being deemed highly relevant to the study [83]-[105]. After completing the search, another highly relevant article came to our attention through a literature information alert system and was also included in the review [106]. In total, 89 articles were reviewed (Figure 1). Articles were evaluated based on the scientific evidence quality scale of the Scottish Intercollegiate Guidelines Network (SIGN) [107]. Table 1 shows the methodological quality of the articles selected. For the specific topics of interest examined in each article, see Table 2.

**Table 1:** **Study design according to SIGN classifying algorithm and SIGN quality of evidence scale**

Study design and quality of evidence	N	%	References
Systematic review or Meta-analyses	21	23.6	
?High quality	8		[40],[42],[46],[61],[62],[65],[71],[72]
?Acceptable	13		[18],[19],[28],[33],[41],[43],[50],[64],[67],[73]-[76]
Economic evaluation	6	6.7	
?High quality	6		[55],[57]-[60],[63]
?Acceptable	0		
Controlled trial	4	4.5	
?High quality	4		[52],[77],[78],[106]
?Acceptable	0		
Cohort studies	18	20.2	
?High quality	11		[45],[69],[86],[87],[89],[90],[93]-[95],[97],[105]
?Acceptable	7		[44],[85],[88],[91],[100],[102],[103]
Case-control study	5	5.6	
?High quality	3		[47],[79],[92]
?Acceptable	2		[39],[99]
Cross-sectional study*	35	39.8	[20]-[27],[29]-[32],[34]-[38],[48],[49],[51],[53],[54],[56],[66],[68],[70],[80]-[84],[96],[98],[101],[104]
Total	89	100	

*The scientific quality of cross-sectional studies was not evaluated because it is not required by the SIGN scale.

**Table 2:** **List of topics within the areas of social, economic, and health impact of respiratory syncytial virus and corresponding references**

Topics		References*
Incidence		[18],[20],[21],[23]-[26],[29],[31],[32],[43],[54],[55]
Prevalence		[23],[70],[75]
Impact on health	Respiratory infections	[21],[25]-[27],[29],[31],[32],[35],[36],[75],[84]
	Wheezing and asthma	[20],[22],[28],[41]-[45],[51],[67],[75],[79],[83],[85]-[95],[105]
	Allergic rhinoconjunctivitis	[41],[45],[79],[95]
	Mortality	[18],[20],[22],[24],[46],[47],[49]-[51],[63],[67],[82]
	Acute otitis media	[51]-[53]
	Apneas	[31],[33],[67],[73]
	Neurological involvement	[48],[73]
	Cardiac involvement	[73]
	COPD	[35]-[40]
	Other	[28],[31],[41],[42],[45],[48],[51],[52],[56],[67],[73],[79],[84]
Risk factors		[20],[21],[41],[47],[70],[80],[103]
Impact on use of resources	Primary care visits	[20],[22],[34],[51]
	Emergency room visits	[22],[34],[51]
	Hospital visits	[22],[34],[41],[51]
	Hospital admissions	[18],[20]-[26],[29],[30],[32],[34],[41],[46],[47],[49],[51],[54]-[56],[63],[65],[66],[77],[78],[80],[81],[96]-[101]
	UCI admissions	[20]-[22],[24],[29],[32],[34],[41],[47],[48],[52],[56],[63],[65],[77],[78],[101]
	Diagnostic tests	[21],[22],[30],[56]
	Ventilation	[22],[24],[29],[31],[48],[49],[56],[63],[77],[78],[101]
	Treatment	[22],[26],[30],[34],[74],[77],[102]
Impact on costs	Direct costs	[34],[41],[51],[55],[57]-[63],[70]
	Indirect costs	[41],[59]-[63]
Strategies to reduce impact	Hygienic measures	[41],[49],[71],[104]
	Prophylactic treatment	[19],[21],[34],[41],[46],[48],[49],[52],[55],[57]-[65],[69],[70],[72],[74],[76]-[78],[105],[106]
Spain		[21],[25],[26],[29],[30],[41],[47],[48],[58]-[60],[63],[64],[77],[80]
Adults		[22],[24],[35]-[40],[50],[54],[75]

*The 23 references obtained from the full reading of the articles selected by the experts on the systematic search were only classified within the topic mentioned in the article [83]-[105], similarly with the article included last [106].

## Results

### Health impact

#### RSV respiratory infections

Population-based incidence studies on RSV infection are scarce, except in children especially in high-risk pediatric population. There were an estimated 33.8 million (95% CI: 19.3-46.2) new diagnoses worldwide of RSV-associated acute lower respiratory infections (ALRI) in children under the age of 5 (22% of total ALRI episodes), with at least 3.4 (95% CI: 2.8-4.3) million cases requiring hospital admission due to severity [18]. Prescott and colleagues reported an incidence of 24 RSV infections per 1,000 live births [19] but higher incidences are common in the presence of prematurity, congenital heart defects, or immunosuppression. Recent studies have reported infection rates of 34.9% among children with preterm birth (gestational age (GA) ≤32 weeks) [20] and 22.5% in children under the age of 2 with severe congenital heart disease admitted for ALRI and/or upper respiratory infections [21].

For all ages, the annual estimate of new RSV infections is 64 million [19]. The annual RSV infection rate in adults has been estimated between 3% and 7% in patients over 65 years of age with no comorbidities and between 4% and 10% in high-risk adults (e.g., with chronic cardiac or lung disease) [22].

ALRI is commonly caused by RSV, either by itself or in combination with other viral or microbial agents. A recent study found RSV in 36.7% of children under the age of 9 suspected of viral respiratory infection, and in 74.6% of those who had been admitted to the hospital [23]. Studies on adults have reported the presence of RSV in between 6.1% and 10.0% of patients hospitalized with respiratory symptoms [22],[24]. RSV infection in community-acquired pneumonia (CAP) pediatric cases varies between 19.8% and 30.5% [25],[26] and it is estimated at about 3.8% in adults [27]. Approximately half of the cases of RSV-induced CAP in children ?3 years of age present with co-infection from other viral agents. Likelihood of co-infection increases with age (36.4% in children ?1 years of age and 65.0% in children between 2 and 3 years of age) [25].

#### Respiratory complications of RSV infection

AB is the most frequent complication derived from RSV infection (85% of children ≤12 month-old and 31% of children between 2 and 5 years of age, hospitalized due to RSV infection [28],[83]). Other studies report that between 61.3% and 77.5% of AB episodes in children younger than 2 years of age are RSV-related [29]-[32].

Although in a majority of AB cases (62%), RSV is the only virus detected [32], the presence of other viruses is not uncommon. The evidence on the role of co-infection on outcomes is inconsistent: whereas some researchers associate co-infection with poorer prognosis [25],[31], others report that co-infection reduces the severity of the condition [32]. Rhinovirus (RV) is the viral agent most frequently associated with RSV (18.1%-26.1% of AB cases with RSV in children ?2 years of age) [31],[32]. More aggressive strategies, such as tracheal or nasotracheal intubation, are required by pediatric AB cases co-infected (RSV?+?RV) (10% of cases) than by those infected with RSV only (7.5% of cases) [31]. In contrast, a study reported that cases of children monoinfected with RSV presented with more severity at admission (42% of RSV monoinfection vs. 31% of RSV?+?other viral infection) [32]. Among infants under 12 months of age admitted for RSV-induced AB or CAP, 79% developed complications, 24% of which were considered serious, the most frequent complication was respiratory (60%). Complications were most frequent in preterm infants between 33 and 35 weeks GA (93%) and with an average age at admission of 2.7 ± 2.5 months [84].

A systematic review on the incidence of apnea in AB episodes among infants reported that rates varied between 0.5% and 37.5% depending on age, preterm birth, comorbidity, and year of the study. For premature infants, rates varied between 4.9% and 37.5%, which is significantly higher than rates among term infants (0.5% and 12.4%). Rates in infants with serious comorbidities vary between 10% and 20.4% versus 1.2% and 4.3% in the absence of comorbidity [33]. The reported incidence of apnea is lower in more recent studies, varying from 20.4% in 1977 to 2.2% in 2006. This likely reflects the improved management of the condition in more recent years [34].

In adults, the prevalence of viral respiratory infection in COPD exacerbations fluctuates between 22.3% and 56% [35]-[40] and its negative impact on lung function and length of hospital stay is greater than that of non-viral flare-ups including a longer recovery time (13 days vs. 6 days) [40]. RSV involvement has been confirmed in 0.7% to 8% of COPD flare-ups [34]-[40]. RSV presence has also been detected in 11% of patients admitted with pneumonia, COPD (11%), heart failure (5%), and asthma (7%) [22].

Research shows that RSV infection during infancy is an independent risk factor for declined lung function (peak expiratory flow rate measured by forced spirometry) in the following years with evidence suggesting a residual impact for as long as 18-20 years when compared to controls (OR: 5.27; 95% CI: 1.60-17.40) [85].

There is evidence of recurrent wheezing episodes associated with a history of RSV infection. The frequency of episodes (44%-48%) may be as high as three times that found among children with no history of RSV infection [41],[42],[86],[87].

RSV-associated AB in children presents very similarly to acute asthma: wheezing, rapid breathing, inflammation of small airways, and, in many cases, respiratory compromise leading to acute lung function failure [83]. In the studies reviewed here we found an association between RSV infection in the early years and childhood asthma [42],[45],[87]-[95]. Most studies follow patients for the first few childhood years and significant associations have been found with relative risk (RR) of 21.8 at a 30-month follow-up [93] and a RR of 5.4 at a 13-year follow-up [95]. The same author published an 18-year follow-up showing that, despite a reduction in RR with age, the association continues to be significant at 18-years (RR: 4.3) [45]. There is also evidence of a significant risk increase in sensitivity to environmental allergens after RSV infection (RR: 1.8) [95].

#### Cardiovascular complications

Cardiovascular complications were found in 9% of infants under 12 months of age admitted for RSV-induced AB or CAP 9% [84]. A systematic review described cardiovascular complications such as atrial and supraventricular tachycardias, life-threatening arrhythmias or cardiogenic shock. Elevated cardiac troponin levels (as expression of myocardial damage) were found in 35% to 54% of infants with RSV infection ventilated in PICUs and have also been found in some children with RSV infection not requiring mechanical ventilation [73].

#### Electrolyte imbalance

About 19% of children under 12 months of age admitted for RSV-induced AB or CAP presents electrolyte imbalance [84]. Hyponatraemia with a serum sodium level of less than 130 mmol/l was found in 11% of infants requiring intensive care with RSV infection; and in 0.6% of children from a less selected population, including patients with milder disease [73].

#### Central nervous system manifestations of RSV

Acute neurological signs and symptoms such as central apneas, seizures, lethargy, feeding or swallowing difficulties, abnormalities of tone or strabism, abnormalities of the cerebrospinal fluid or the electroencephalogram were found in 39% of RSV-positive patients on a PICU. In patients with milder disease neurological complications were found in 1.2% of patients. Looking at the occurrence of seizures as a manifestation of an encephalopathy, another group found an incidence of seizures of 1.8% in patients with RSV bronchiolitis admitted to a paediatric tertiary referral centre [73].

#### Acute otitis media and allergic rhino-conjunctivitis

Acute otitis media (AOM) is often associated with RSV infection. Between 20% and 41% of children admitted with a primary diagnosis of RSV infection (AB or CAP) also present with AOM [51],[52]. Another study reported RSV infection present in 56% of AOM pediatric cases hospitalized between 3 and 18 months [53]. In children admitted for RSV infection in their first year of life, the risk of developing allergic rhino-conjunctivitis was significantly greater than among controls (39% vs. 15%) [41],[45],[95].

#### Mortality

The 2005 worldwide estimates of mortality caused by RSV-associated ALRI in children under the age of 5 range between 66,000 and 199,000 deaths, with 99% of these deaths occurring in the developing world [18]. In developed countries, the contribution of RSV infections to mortality in children, including preterm children, is almost negligible but only among those without associated complications.

A recent meta-analysis [46] calculated preterm infant mortality at 0.04% and slightly higher (0.62%) for those with BPD. A cohort study of 202 preterm children of 32 to 35 weeks GA who were hospitalized for RSV infection observed no deaths [47]. In another study of 1,583 preterm children ?32 weeks GA admitted with RSV infection, one death was observed [48]. However, as a study on nosocomial infection-related mortality among children admitted to ICU reported, when RSV infection is present in children with serious comorbidities, mortality may exceed 25% [49]. In adults and elderly, mortality rates mirror those of influenza and range between 4 and 8% [22],[24],[50] unless in immunosuppressed individuals (e.g., leukemia or transplant patients) where RSV-associated mortality may reach 30-70% [50].

### Impact on use of resources

#### Primary care visits

It has been estimated that 2.2% (1.7 million visits) of all U.S. primary care visits of children ?5 years of age in the year 2000 were caused by RSV infection [51]. Other studies confirmed that RSV-infected children pay a significantly greater number of visits to the doctor than their uninfected counterparts [20],[34]. RSV-infected preterm children 32-35 weeks GA have an average of 12.4 doctor visits during their first two years of life (median?=?12; range?=?1-27) for any cause and 5 visits on average (median?=?4; range?=?0-14) for respiratory causes, whereas the same data for children with no respiratory pathology were 9.4 (median?=?8; range?=?0-34) and 2.9 (median?=?2; range?=?0-17), respectively [34]. A multicenter Spanish study reported that 18.8% of first AB episodes in children under the age of 2 were diagnosed in primary care [30]. A study comparing RSV infection in patients over the age of 65 with no comorbidity and in high-risk patients observed a significantly higher primary care resources usage by high-risk patients. Specifically, 15% of the first group called the doctor and 17% went to the primary care office vs. 23% and 29% for the high-risk group, respectively [22].

#### Visits to specialists

RSV-infected patients also visit a specialist more often, especially high-risk patients with cardiopulmonary disease. In the U.S., it is estimated that 3.2% of visits to specialists among children ?5 years of age are caused by RSV [51]. Further, in RSV-infected preterm children 32-35 weeks GA visits to specialist were estimated 18.4 ± 10.6 vs. 7.5 ± 4.3 for healthy controls [41].

#### Emergency room visits

During their first two years of life, U.K. RSV-infected preterm children 32-35 weeks GA visit the emergency room an average of 3.0 times (median?=?3; range?=?1-6) for any cause and 1.6 times (median?=?2; range?=?1-4) for respiratory causes. These estimates are significantly lower in the absence of respiratory disease (mean?=?0.7 (median?=?0; range?=?0-9) and mean?=?0.1 (median?=?0; range?=?0-7), respectively) [34].

A multicenter Spanish study reported that 51.7% of all first AB episodes in children <2 years of age were seen at the emergency room [30]. In contrast, only 9% of high-risk adults with RSV infection went to the emergency room and no adults over 65 years of age and comorbidity-free were seen in emergency rooms [22].

#### Hospital admissions

Admission rates in RSV-infected children vary substantially by age and comorbidity. Findings in this review varied between rates of 37.0 and 142.8 per 1,000 among preterm children ?32 weeks GA [20],[47],[52],[96]; 57.2 and 138.0 per 1,000 in preterm children 29-32 weeks GA [48],[96]; 23.5 and 29.5 per 1,000 in term children [96],[54]; and 1.8 per 1,000 in 1-4 year-olds [54]. In children ?2 years of age with congenital heart disease, admission rates ranged between 38 and 80 per 1,000 [23],[55]. A 2008 study reported that co-infection with two or more viruses increased the risk of admission up to 67.2% in children with pneumonia vs. 46.1% in the absence of co-infection [25]. Additional factors likely to increase the risk of admission three- and four-fold include: prematurity, low birth weight, immunosuppression, BPD, neurological issues, being ?10 weeks at the start of RSV season, breastfeeding under 2 months, school-age siblings, going to daycare, living with at least 4 adults, exposure to environmental pollution or tobacco smoke, maternal tobacco use during pregnancy, and family history of asthma [20],[21],[41],[47],[97]-[99]. Risk increases substantially in the presence of two or more of these factors [47]. Lower admission rates have been reported in the general population (children and adults) (0.553 per 1,000; 95% CI: 0.44-1.06) [54], although rates vary considerably based on comorbidity. Admission rates between 15 and 16% have been reported in high-risk adults with RSV infection [22],[24].

The number of hospitalization days due to RSV varies substantially by age of the patient and country of residence. Findings in the studies reviewed ranged from 1.2 to 15 inpatient days [20]-[22],[24],[26],[29],[31],[32],[41],[47],[49],[56],[100],[101]. In children 32-35 weeks GA, the median duration was 7 days (interquartile range?=?4-10 days) [47]. In children 29-33 weeks GA the average number of days at the hospital was 7.4 ± 4.9 days and, in those 33-36 weeks GA, the average is 7.9 ± 6.0 days [100]. Another study reported similar data: 6.8 days for children ?32 weeks GA and 8.4 days for those 33-35 weeks GA [101].

In the last 10 years in Spain, hospital stays for children <2 years of age with or without related risk factors averaged 5-8 days [21],[29], in contrast with an average of 1.2-3.8 days in the U.S. [56] and U.K. [20]. In children under 14, average stay was similar before 18 months (5.7 days) and after 18 months of age (5.8 days) [26]. Data examined on adults also show substantial fluctuation, varying from 3 days (range?=?2-6 days) to 14 ± 8 days [22],[24].

The current evidence on the role of co-infection on the length of hospital stay is inconclusive. Some studies found that co-infection increased the length of stay (53.7% vs. 47.6% stayed ?3 days; p?<?0.05) [31], whereas others found that co-infection shortened it (47.8% vs. 57.4% stayed in the hospital ?4 days; p?<?0.02) [32].

#### ICU admissions

Intensive Care Unit (ICU) admissions among those hospitalized due to RSV infection also vary according to age, comorbidity, and country. Premature birth is also a risk factor for ICU admission, especially in infants 33-35 weeks GA for whom admission rates varied between 17.8% and 48.4% [47],[101]. In Spain, 1.5% of children <2 years of age with AB needed ICU admission, 3.6% in Israel [32] and between 11.6% and 17.5% in the U.S. [30],[31],[56]. In Spain, about 30.4% of children ?2 years of age admitted for congenital heart disease and RSV infection required ICU admission [21]. In U.S. adults, admissions varied between 9.7% [24] and 15% in high-risk adults [22].

The mean and median stay in ICU for children under the age of 2 was about 4 days [56],[101], although it reached a median of 10 days (range?=?5-18 days) if the patient presented with congenital heart disease [21]. In the case of prematurity, the average stay was 7.7 days for those 33-35 weeks GA and about 6 days for those ?32 weeks GA [48],[101]. In adults over the age of 50, the median stay reported was 5 days (range?=?3-12 days) [24].

#### Diagnostic tests for RSV infection and its clinical management

A multicenter study including 5,647 children <2 years of age with a first AB episode, reported that oxygen saturation was measured in 73% of patients, chest X-ray was performed in 31.3%, blood gases were measured in 11.8%, blood count in 22.2%, and RSV tests were performed in 37.4%, of which 63.5% turned out positive.

Tests were significantly more likely to be performed if the child was younger [30]. In the U.S., it was estimated that children ?5 years of age with RSV-induced respiratory infection received chest X-rays in 20%, 45%, and 7% of cases when seen in primary care, emergency room, and hospital outpatient visits, respectively [51]. Other tests frequently performed in emergency rooms included oxygen saturation (36%) and blood count (20%) [51]. Also in the U.S., adults with RSV-induced respiratory infection received chest X-rays in 7% of elderly with no comorbidity and 20% of high-risk adults [22].

### Treatments

In patients ?2 years of age with AB, oxygen was required by the 22.1% [30]. Among those hospitalized, oxygen was used more frequently in the presence of RSV infection than in its absence (56.3% vs. 46.7%) [56].

Between 6% and 18.3% of children hospitalized for RSV infection required assisted ventilation [47],[49],[56] for a median period of time estimated at 6 days (range?=?4.13) [48],[56]. Between 3.2% and 13% of adults required assisted ventilation [22],[24]. The need for intubation in pediatric cases admitted for AB or RSV pneumonia varied according to GA. For infants ?32 weeks, 33-35 weeks, and ?37 weeks GA, intubation was required in 21.4%, 8.7%, and 12.1% of cases, respectively [101]. Co-infection increased the risk of intubation in children ?2 years of age with RSV-induced AB (10% vs. 7.5% for not co-infected children) [31].

The use of antibiotics varied according to age and presence of comorbidity. The 13.6% of AB patients ?2 years of age received antibiotics [30]. In patients with RSV infection as the only respiratory infectious agent, antibiotics were administered to 24.9% of children ?18 months old and 46.8% of children over 18 months old [26]. In the U.S., whereas only 9% of elderly patients with no related comorbidity received antibiotics, its administration reached 43% among high-risk adults [22].

The 64.7% of AB patients ?2 years of age were treated with short acting inhalers of ?_2-_ adrenergic agonist [30]. However, U.S. studies report lower usage, with only 4% of healthy elderly and 29% of high-risk adults receiving this treatment [22].

Corticoids were administered to children with RSV-induced AB orally (16.8%), via inhalation (4.8%), or parenteral (5.8%) [30]. There were reports of 21% of RSV-infected high-risk adults receiving parenteral corticoids [22]. It is possible that part of the of antibiotics, corticoids, and bronchodilators prescription may be due to overuse by lack of adherence to clinical practice guideline recommendations.

The 27.6% of AB pediatric cases required antipyretics [30]. Higher usage has been reported in RSV-infected adult patients over 65 years of age with no comorbidity (46%) than in high-risk adults (32%) [22].

### Economic impact

The economic studies reviewed were mostly focused on assessing the effiency of palivizumab for the prophylaxis of RSV infection. Assessments were based on a decision-making model based on the existing literature [55],[57]-[60], analyses of a U.S. nationally representative database [51], or systematic literature reviews [61]-[63]. Four of the studies examined contributed data on direct costs (DC) for years 2000, 2005 and 2006 [51],[58]-[60], and three provided data on indirect costs (IC) for years 2005 and 2006 [58]-[60].

### Direct and indirect costs

Direct costs of an AB episode in infants ?35 weeks GA have been estimated at 466€, and at 2,134€ when including subsequent recurrent wheezing episodes [58]. In infants <32 weeks GA, costs increased as GA decreased. DC estimates reached 1,169€ per acute episode and 5,608€ when also treating recurrent wheezing [59]. A 2006 study estimated the DC of acute episodes at 421€ in children 32 to 35 weeks GA with two associated risk factors [60]. Finally, in the U.S., the hospitalization costs per child ?5 years of age with RSV were estimated at $4,584 [60]. Corresponding indirect costs estimated in these three studies for children not on palivizumab prophylaxis were: 3.159€ [58], 4.634€ [59], and 1.768€ [60].

### Impact on quality of life

Recurrent wheezing after RSV-induced AB negatively impacts children's quality of life [103]. The authors measured quality of life with a validated questionnaire with 13 dimensions and found a negative impact not only in the respiratory system dimension, but also in the one related to gastrointestinal tract and sleep quality. Quality of life is worse during the winter months, when wheezing episodes tend to increase.

### Strategies to reduce the impact of RSV infection

#### Social factors

Home overcrowding (?4 adults), school-age siblings, going to daycare, exposure to tobacco smoke, and breastfeeding period shorter than two months are some of the risk factors for RSV infection requiring hospital admission [20],[47],[103]. Tobacco smoke exposure is also a risk factor for increasing the length of hospital stays [20].

#### Hygienic measures

Parents must be informed of the basic hygienic habits that help prevent spreading RSV infection, especially to highly vulnerable preterm infants. Frequent hand washing and isolation of RSV-infected patients have proven effective means of reducing RSV nosocomial RSV infections [104].

#### Prophylactic treatment

Palivizumab is the only approved drug to prevent RSV-induced ALRI and minimize complications. The study IMpact-RSV observed a 55% (95% CI: 38%-72%) reduction in hospitalizations after prophylaxis with palivizumab (Number Needed to Treat, NNT?=?17). The efficacy was higher in children without BPD (78%; 95% CI: 66%-90%) (NNT?=?16) than in those with BPD (38%; 95% CI: 20%-58%) (NNT?=?20) [52],[64]. In children with congenital heart disease, palivizumab prophylaxis reduced RSV-related hospital admission rates between 58% and 86% [64]. Other studies have reported reductions in ICU [64],[65] and hospital [66] admission rates around 50% due to palivizumab prophylaxis.

In addition, a substantial reduction in self-reported incidence of recurrent wheezing was observed in infants ?35 weeks GA who received palivizumab prophylaxis when compared to controls (13% vs. 26%) as well as in doctor-diagnosed incidence (8% vs. 16%). Prophylaxis was also associated with a delay in the onset of wheezing [105]. A recent clinical trial involving healthy preterm infants 33-35 weeks GA treated for immunoprophylaxis with palivizumab reported a relative risk reduction (RRR) of 47% (95% CI: 14%-80%) in the proportion of children with recurrent wheezing episodes and a reduction of 61% (95% CI: 56%-65%) in the total number of days with wheezing episodes during the infant's first year [106].

The cost-effectiveness of RSV infection prophylaxis with palivizumab varies substantially depending on the patient group. A substantial portion of effectiveness studies reviewed concluded that preterm infant prophylaxis, especially in those with associated risk factors, was cost-effective in terms of the cost per Quality Adjusted Life Years (QALY) gained [57]-[60],[62]. However, other authors did not find it cost-effective in terms of costs per avoided hospital admission [55],[63]. Regardless, the cost-effectiveness ratio was strongly associated to the mortality rate of the reference group [61]. According to the model proposed by Mahadevia et al. [57], prophylaxis was cost effective in preterm infants 32-35 weeks GA and chronological age (CA) ?6 months, with associated risk factors. Effectiveness improved in preterm infants 32-34 weeks GA and CA ?3 months and, especially in children ?32 weeks GA and CA ?6 months. Lázaro and colleagues [58] found an incremental cost-effectiveness relationship (ICE) for medical direct costs, including recurrent wheezing, of 13,846€ per QALY (12,208€/QALY for preterm infants and 17,620€/QALY for chronic lung disease (CLD)). In all cases, QALY was well under the 30.000 €/QALY threshold which is the generally socially acceptable standard in Europe. The cost-effectiveness ratio was more favorable when indirect costs were included. Thus, authors concluded that palivizumab was a cost-effective treatment to protect high-risk children against RSV.

## Discussion

For the most part, available population-based data on RSV are estimates based on analyses of administrative databases of hospital admissions or on review studies of specific age groups or clinical condition. Incidence data may be underestimated, however, both in children and adults. For pediatric cases, for instance, where RSV infection is most common, the majority of clinical guides for AB management do not recommend the routine use of diagnostic tests since diagnosis does not affect treatment and only a small number of patients require admission. Still, in hospitalized children, diagnosis may be of interest in order to establish hospital cohorts when isolation of the child is not feasible [108], or for epidemiological reasons.

RSV infection is rarely diagnosed in adults because the clinical presentation tends to be non-specific and easily mistaken for an influenza outbreak. Further, physicians avoid requesting the more complex diagnostic tests required for adults such as the RT-PCR (*reverse transcription polymerase chain reaction*) and/or blood tests [22],[50]. Despite the potential underestimation bias, the incidence rate found in studies such as Falsey et al. is relevant being twice that of influenza A, with a similar disease burden in terms of hospital stays and mortality in high-risk adults [22].

The clinical presentation of RSV infection in children and adults varies significantly. In children the most common presentation is AB. In full term children without comorbidities the presentation tends to be benign and resolves in a few days, although between 2% and 3% do require admission. In preterm children or with cardiorespiratory comorbidity, the risk of hospitalization as well as complications, especially respiratory ones such as apnea, increase substantially and even full term and comorbidity-free children frequently suffer AOM as a complication.

In healthy adults the clinical presentation for RSV may mirror that of influenza, with similar consequences. RSV infection has often been involved in exacerbations of COPD. The importance of preventing viral infection in these patients is based on avoiding the greater physical decline, greater hospitalization rates, and longer recovery time that viral exacerbation episodes bring compared to the exacerbations caused by bacterial infections [40].

The childhood mortality risk associated to RSV infection is almost negligible, except in children with severe associated morbidity for whom 25% mortality rate has been reported. Mortality rates in adults and the elderly are similar to that of influenza, except in hospitalized populations with mortality rates up to 80% [22].

The use of diagnostic and treatment resources for RSV infection acute episodes contribute substantially to the disease's burden on healthcare costs. According to findings from a multicenter study by González de Dios and colleagues [30] the most frequently performed diagnostic tests on children are: oxygen saturation (3 out of 4 children), RSV rapid diagnostic test and chest X-ray (one third of children), and blood count (1 out of 5 children). However, the study failed to specify where the tests were performed. Based on other studies, one could assume that most of the tests were performed in the emergency room although a large portion of the chest X-rays may have been prescribed in primary care [51].

Data on diagnostics and treatment for RSV infection in adults are scarce in general and non-existent for Spain. Based on the limited information found we concluded that chest X-ray is a common diagnostic tool, especially among patients with cardiopulmonary comorbidity [22].

Over half of children admitted with RSV-induced respiratory infection require oxygen therapy. And a significant number (between 1 and 5 in 10) require assisted ventilation due to recurrent apneas, hypoxemia, increased work of breathing, and respiratory failure [47],[49],[56]. The need for intubation is greater in children, especially preterm infants (1 in 5 infants ?32 GA is intubated) or co-infected with RV (1 in 10???2 years of age). Unfortunately, there is lack of consensus regarding the best type of ventilatory support [67].

Estimating the use of oxygen therapy in adults due to RSV infection is a difficult task because in many cases the patients had COPD and were already using this therapy. Studies report that a substantial proportion of these patients (up to 1 in 10) required assisted ventilation [22],[24].

Although RSV infection is viral, antibiotics are often used even in the absence of bacterial co-infection. In Spain, one fourth of pediatric patients ?18 months and half of those >18 months received antibiotics. No data on Spain adults were found, but in the U.S. antibiotics were prescribed to half of the high-risk patients and to 1 in 10 patients with no related comorbidity.

The use of other drugs such as bronchodilators was fairly common, especially in children. In Spain, they were used by two thirds of children ?2 years of age with RSV-induced AB [30]. In the U.S., thirty percent of high-risk adults were treated with bronchodilators [22]. Although the effectiveness of corticoids is not proven in children with AB [67], their use is not uncommon in Spain, especially administered orally and, to a lesser extent, through inhalation or parenterally (1 in 20) [30]. In the U.S., 1 in 4 high-risk adults are treated with parenteral corticoids [22].

The impact of RSV on medical resources goes beyond hospital admissions, diagnostic tests, and treatments necessary to manage infection-related episodes. For instance, the volume of follow-up primary care visits following RSV infection is significant [22],[51]. Data are likely to be underestimated, however, due to lack of accounting of visits related to medium- and long-term respiratory complications. Recent findings by Shefali-Patel et al. [34] suggest that primary care visits during the two years following the first RSV infection-related episode in infants 32-35 weeks GA almost doubled that of controls. One of the potential complications of RSV infection are abnormalities in the spirometric flow which are 5 times more likely in children with a history of RSV infection than in controls and which risk may not subside for 20 years after the initial episode [85]. Other potential complications are recurrent wheezing (3 times more likely than in controls [41],[42],[86],[87]) and asthma, with a 20-fold risk during the first few months post initial episode compared to controls [93]. Although the risk of asthma decreases with time, it remains four times higher than in controls even after reaching adulthood [45]. Evidence on the causal relationship between RSV infection and asthma remains inconclusive. For some authors, the greater sensitivity to environmental allergens after RSV infection may be explained by having common risk factors, such as genetic predisposition. This hypothesis finds support in a twin study that concluded that the association between severe RSV infection and asthma was bidirectional [68]. However, studies using prophylactic treatment of RSV with palivizumab have shown a 50% reduction in recurrent wheezing rates compared to controls [69],[105],[106] which suggest a causal relationship between RSV infection and asthma. Finally, another common medium-term complication, such as allergic rhino-conjunctivitis, may also contribute to the greater primary care usage.

RSV infection in children also has a substantial impact on visits to the specialist. Visits more than double in preterm children and those with cardiorespiratory disease [41]. Regarding emergency services, Shefali-Patel and colleagues found that children with a history of RSV infection are over three times more likely to use the emergency room in the two years following infection than those never infected [34]. In the U.S., a third of high-risk adults and one fifth of those without comorbidities reported visiting a specialist due to RSV infection. In contrast, visits to the emergency room were relatively low (one in 10 high-risk adults and none among adults with no comorbidities) [22].

Data collected here on hospitalization rates and length of stay in children infected with RSV vary substantially not only due to the difference among age groups and comorbidity levels but also due to the differing characteristics of healthcare systems across countries. For instance, estimating the hospitalization rate for all ages from studies performed in Spain is problematic. Results from a survey of Spanish hospitals, emergency services, primary care centers, and doctor offices [30] show that 34.6% of over 5,000 AB episodes were admitted, that RSV diagnostic tests were performed in one third of all episodes, and that 63% were positive for the virus. However, the method of collecting and reporting data in Spain prevents the estimation of hospitalization rate due to RSV. Carbonell and colleagues [48] reported a 13% admission rate for children <32 GA, the highest rate reported in children with this GA. This result, when combined with Spanish data on average length of hospital stay due to RSV infection in children <2 years of age (5-8 days) [21],[29], is more than twice that reported in U.S. or U.K. [20],[56] and suggests that the Spanish admission rate may be one of the highest reported.

In the U.S. rates also vary by comorbidity levels and estimates show that one in six of high-risk adults are admitted [22]. Although no other data are available, that we are aware of, we would have expected higher rates.

In Spain, ICU admission is rare for children with no comorbidities but it may be necessary for almost one third of those with congenital heart disease hospitalized with RSV infection, a comorbidity that may extend the length of stay from 4 to 10 days [21]. In U.S. adults, one in six high-risk patients required admission to the ICU [22], and for patients >50 years of age the average ICU stay was 5 days [24].

Estimated DC found in the literature support the information we found on healthcare resources dedicated to treating the acute phase of RSV infection episode in children as well as the mid- to long-term complications. Average DC to treat the acute episode is four times lower than the costs related to the following recurrent wheezing episodes, regardless of the child's age [58],[59]. From a social perspective indirect costs should be taken into account, which are similar to the costs related to the sequelae of recurrent wheezing episodes according to the studies reviewed [58],[59].

To our knowledge, there are no cost-effectiveness studies examining the economic burden of RSV infection. In contrast with the negligible RSV-related child mortality, mortality in hospitalized adults may reach 8% [22], an important factor to consider when estimating the economic burden of the disease. Another understudied aspect is the medium- and long-term consequences with or without COPD. Thus, the costs and real economic impact of RSV infection in adults, especially high-risk adults, is an area in need of further research.

Interventions to prevent RSV infection are unable to encompass all the social risk factors. Several of these factors are not easily amenable to change such as the number of adults living in the household, siblings attending school, or daycare attendance. However, interventions could increase general knowledge on RSV infection mechanisms and factors such exposure to tobacco smoke, breastfeeding duration, and hygienic measures, as well as the management of high-risk patients.

The effectiveness of palivizumab in reducing admission rates, ICU length of stay, and morbidity in children with or without related risk factors has been demonstrated [52],[64]. However, its high cost and the almost negligible children mortality has limited its use to treat children with risk-factors [70]. Estimates derived from effectiveness models in preterm high-risk infants are under the accepted threshold of 30,000€/QALY, thus, the use of palivizumab falls within the socially acceptable range of financing by public health care systems [58]-[60]. However, no studies have been found on the cost effectiveness of prophylactic treatment among high-risk adults despite two important factors affecting this population: a. mortality rates are not negligible; and b. the burden of disease as a result of the medium- and long-term consequences of RSV infection is likely to be as substantial as for high-risk children.

## Conclusions

Based on the results of this review, we conclude that:

- The health impact of RSV infection is significant and goes beyond the acute episode phase.
- The health impact of RSV infection on children is much better documented than on adults.
- Further research is needed on the medium- and long-term impact of RSV infection on the adult population, especially those high-risk.
- There is a need for interventions aimed at reducing the impact of RSV infection by targeting health education, information, and prophylaxis in high-risk populations.

## Authors’ original submitted files for images

Below are the links to the authors’ original submitted files for images. Authors’ original file for figure 1
