# Supplementary figures and images for: Social, economic, and health impact of the respiratory syncytial virus: a systematic search
Source: BMC Infect Dis. 2014 Oct 30;14:544. doi: 10.1186/s12879-014-0544-x (PMC4219051; doi:10.1186/s12879-014-0544-x)

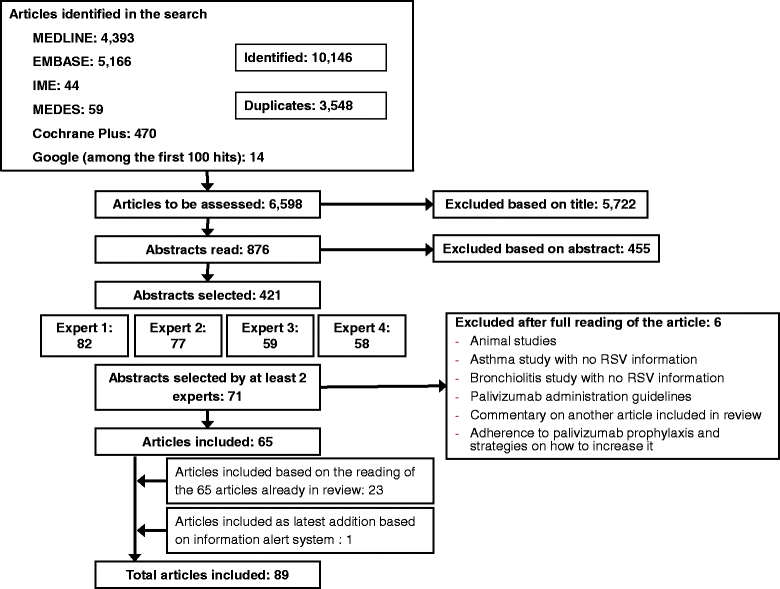

Supplement: Supplementary file 1 — Authors’ original file for figure 1 [file 12879_2014_544_MOESM1_ESM.gif]
